# Plant Defense Mechanisms against Polycyclic Aromatic Hydrocarbon Contamination: Insights into the Role of Extracellular Vesicles

**DOI:** 10.3390/toxics12090653

**Published:** 2024-09-05

**Authors:** Muttiah Barathan, Sook Luan Ng, Yogeswaran Lokanathan, Min Hwei Ng, Jia Xian Law

**Affiliations:** 1 Department of Tissue Engineering and Regenerative Medicine, Faculty of Medicine, Universiti Kebangsaan Malaysia, Cheras, Kuala Lumpur 56000, Malaysia; lyoges@ppukm.ukm.edu.my (Y.L.); angela@ppukm.ukm.edu.my (M.H.N.); 2 Department of Craniofacial Diagnostics and Biosciences, Faculty of Dentistry, Universiti Kebangsaan Malaysia, Jalan Raja Muda Abdul Aziz, Kuala Lumpur 50300, Malaysia; ngsookluan@ukm.edu.my

**Keywords:** polycyclic aromatic hydrocarbons (PAHs), environmental contamination, plant stress responses, plant-derived extracellular vesicles (PDEVs), phytoremediation, oxidative stress

## Abstract

Polycyclic aromatic hydrocarbons (PAHs) are persistent organic pollutants that pose significant environmental and health risks. These compounds originate from both natural phenomena, such as volcanic activity and wildfires, and anthropogenic sources, including vehicular emissions, industrial processes, and fossil fuel combustion. Their classification as carcinogenic, mutagenic, and teratogenic substances link them to various cancers and health disorders. PAHs are categorized into low-molecular-weight (LMW) and high-molecular-weight (HMW) groups, with HMW PAHs exhibiting greater resistance to degradation and a tendency to accumulate in sediments and biological tissues. Soil serves as a primary reservoir for PAHs, particularly in areas of high emissions, creating substantial risks through ingestion, dermal contact, and inhalation. Coastal and aquatic ecosystems are especially vulnerable due to concentrated human activities, with PAH persistence disrupting microbial communities, inhibiting plant growth, and altering ecosystem functions, potentially leading to biodiversity loss. In plants, PAH contamination manifests as a form of abiotic stress, inducing oxidative stress, cellular damage, and growth inhibition. Plants respond by activating antioxidant defenses and stress-related pathways. A notable aspect of plant defense mechanisms involves plant-derived extracellular vesicles (PDEVs), which are membrane-bound nanoparticles released by plant cells. These PDEVs play a crucial role in enhancing plant resistance to PAHs by facilitating intercellular communication and coordinating defense responses. The interaction between PAHs and PDEVs, while not fully elucidated, suggests a complex interplay of cellular defense mechanisms. PDEVs may contribute to PAH detoxification through pollutant sequestration or by delivering enzymes capable of PAH degradation. Studying PDEVs provides valuable insights into plant stress resilience mechanisms and offers potential new strategies for mitigating PAH-induced stress in plants and ecosystems.

## 1. Introduction

Polycyclic aromatic hydrocarbons (PAHs) are persistent organic pollutants with significant carcinogenic, mutagenic, and teratogenic effects, originating from both natural and anthropogenic sources [1]. The persistence of PAHs in soils is a critical issue; their complex structures and low volatility lead to a prolonged environmental presence and accumulation in soil matrices [2]. For instance, compounds like benzo[a]pyrene, a common PAH, are highly resistant to degradation and can remain in soils for decades. The behavior of PAHs in soil systems is complex. They tend to adsorb on to soil particles and organic matter, reducing their mobility but increasing their potential for bioaccumulation in plants and animals [3]. This bioaccumulation can lead to significant health risks as PAHs move up the food chain, ultimately impacting human health through dietary exposure [4]. Studies have shown that chronic exposure to PAHs can result in serious conditions such as cancer, respiratory disorders, and developmental issues. For example, elevated levels of PAHs in agricultural soils have been linked to increased incidence of cancer in populations consuming contaminated crops [5]. PAHs pose long-term environmental risks, leading to bioaccumulation in the food chain and adverse effects on plants, animals, and humans, including reproductive issues, immune suppression, cancer, and neurotoxicity [6]. Given these concerns, understanding the fate and transport of PAHs in soils, as well as their potential environmental and health impacts, is essential for developing effective mitigation strategies. 

Plant-derived extracellular vesicles (PDEVs) are nano-sized membrane-bound particles secreted by plant cells into the extracellular space. These vesicles, which include exosomes, microvesicles, and apoptotic bodies, play crucial roles in intercellular communication and the transfer of molecular cargo such as proteins, lipids, RNA, and metabolites. PDEVs offer a novel approach for PAH remediation. These EVs can facilitate the biodegradation and detoxification of PAHs, enhance the bioavailability of detoxifying compounds, and aid in the adsorption and sequestration of PAHs in soil and water. 

The aim of this review article is to evaluate the potential of PDEVs for the remediation of PAHs. It seeks to explore how these EVs can enhance PAH degradation, improve detoxification, and facilitate the adsorption and sequestration of PAHs in contaminated soils, while also comparing their effectiveness with traditional remediation methods and identifying future research directions. The hypothesis of this review article is that PDEVs can significantly enhance the bioremediation of PAHs in contaminated soils. This hypothesis is based on the premise that PDEVs possess enzymatic and bioactive molecules capable of degrading PAHs into less harmful forms, improving the bioavailability of detoxification compounds, and facilitating the adsorption and sequestration of PAHs, thereby aiding in the remediation of PAH contamination. The review is structured to first provide an overview of PAHs, including their sources, classification, and characteristics. It then discusses the analytical techniques used to detect and measure PAHs, highlighting advancements in technology for improved sensitivity and accuracy. Following this, the review examines the environmental and health impacts of PAHs, focusing on their persistence in soil, their bioaccumulation in the food chain, and the associated risks to ecosystems and human health. The discussion extends to the specific challenges faced in coastal and Arctic regions due to PAH pollution. Finally, the review concludes with a summary of current regulatory measures and the need for ongoing monitoring to address the persistent threats posed by PAHs.

## 2. Polycyclic Aromatic Hydrocarbons (PAHs) 

PAHs are persistent organic pollutants known for their colorless, white, or pale-yellow appearance. PAHs are released into the environment both from natural and anthropogenic sources, including volcanic eruptions, forest fires, vehicle emissions, industrial processes, and the burning of fossil fuels and biomass [1]. These compounds are garnering significant attention due to their classification as carcinogenic, mutagenic, and teratogenic substances. Studies have reported that exposure to PAHs has been linked to a range of health problems, including lung cancer, melanoma, and bladder cancer [2]. PAHs can be categorized into two groups: low-molecular-weight (LMW) PAHs, which contain 2–3 rings, and high-molecular-weight (HMW) PAHs, which consist of 4–7 rings [3,4,5,6]. A total of 200 different kinds of PAHs exists and, as the number of benzene rings in PAHs grows, their chemical and physical characteristics exhibit anticipated alterations [4]. These bigger PAHs have lower volatility, decreasing their ability to evaporate into the atmosphere. Additionally, their solubility in water decreases, causing them to have a higher likelihood of gathering in sediments and biological tissues such as plants instead of dissolving in water [7]. Moreover, the greater structural complexity of these larger PAHs causes them to be more resilient to degradation by natural processes or biological systems. At the same time, their increased ability to dissolve in fats also makes them more likely to stick to particles, such as soil, dust, and organic material in water systems [3,7]. Together, these characteristics enhance the likelihood of larger PAHs persisting and accumulating in the environment.

Some of the common PAHs are naphthalene, acenaphthylene, acenaphthene, fluorene, phenanthrene, anthracene, fluoranthene, pyrene, benzo[a]anthracene, chrysene, benzo[b]fluoranthene, benzo[k]fluoranthene, benzo[a]pyrene, indeno [1,2,3-cd]pyrene, dibenz[a,h]anthracene, and benzo[g,h,i]perylene [4]. Due to their toxicity and environmental persistence, all these 16 PAHs have been prioritized for monitoring by the European Union and the United States Environmental Protection Agency. Their persistence in the environment coupled with their ability to bioaccumulate makes them pose significant risks to human health—such as Alzheimer’s disease (AD), cardiovascular diseases (CVD), and chronic obstructive pulmonary disease (COPD)—as well as ecosystems, thus making them subject to environmental monitoring as well regulatory measures [8,9,10].

High-performance liquid chromatography (HPLC) and gas chromatography (GC) are powerful tools for analyzing PAHs [3]. HPLC, often paired with fluorescence or ultraviolet detectors (HPLC-FL/UV), excels in PAH detection. GC, particularly when coupled with mass spectrometry (GC-MS), boasts even higher sensitivity. Recent advancements, such as LC-MS/MS (tandem mass spectrometry) and GCxGC (comprehensive two-dimensional GC), have further enhanced PAH analysis in complex environmental samples like soil, sediment, and biota [11]. These advancements have achieved even lower detection limits and improved accuracy [9].

## 3. Soil as a Critical Reservoir

PAHs are transported long distances to remote ecosystems like high mountains, the Arctic, and Antarctic. Biological sources also contribute to soil PAH levels. Soil plays a crucial role as a major environmental storage site for PAHs, making up more than 90% of their content in ecosystems [12], especially in countries that are still developing, such as China, which saw a total of 35,289 tons of PAH emissions in 2020 [13]. Benzo[a]pyrene (BaP) emerged as the most significant contributor to the total health risks associated with the 16 PAHs analyzed [14]. A study has found that the BaP content in the green parts of *Hordeum sativum* increased from 0.3 µg/kg in control soil to 2.6 µg/kg and 16.8 µg/kg under BaP concentrations of 20 and 400 ng/g, respectively. Similarly, in the roots, BaP concentrations increased from 0.9 µg/kg in control soil to 7.7 µg/kg and 42.8 µg/kg under the same conditions [15]. Meanwhile, a BaP concentration threshold of 0.24 mg/kg was calculated, which is over 10 times higher than the limit established in Spain [16]. An exceedance of BaP standards in soils and road dust indicates a hazardous environmental situation in Alushta, Yalta, and Sebastopol, with PM10 particles being particularly dangerous due to their potential for forming anomalies with extreme levels of BaP contamination [17]. Meanwhile, BaP concentrations in municipal soils in Cleveland, USA have been reported between 0.28 and 5.50 mg/kg, indicating significant pollution levels in this urban environment [18]. Historical data from 1993 to 1994 show BaP concentrations ranging from <0.0001 to 3.030 mg/kg, with higher levels associated with motor vehicles and residential furnaces in Poland. A later study in Bialystok reported BaP levels from 0.3 to 0.9 mg/kg in soils [19]. In Turkey (Antalya Aksu region), BaP levels in soils from greenhouse crops were lower, with a mean value of 2.31 µg/kg [20]. Urban soils had the highest average concentration (169.59 ng/g, range 41.29–295.90), agricultural soils had a lower average (119.48 ng/g) but a wider range (8.23–955.05 ng/g), and montane soils had the lowest levels (mean 33.63 ng/g, range 13.32–79.74), likely due to distance from pollution sources [21]. Similarly, BaP concentration in the surface soil horizons (0–10 cm) was found to be 409 ng/g. This level is significantly elevated, being 83 times higher than the local background value and 20 times above the maximum permissible concentration (MPC) accepted in Russia [22]. In Bursa, Turkey, BaP deposition in soil samples from the same region was analyzed and the study reported total PAH concentrations ranging from 13 to 189.4 ng/g dry matter (DM). The study also noted that PAH concentrations were elevated during the colder months, indicating a seasonal influence on contamination levels [23]. In Zhiwu Park during the summer, the concentration of BaP was recorded at 0.944 mg/kg. This level significantly exceeds the screening value of 0.55 mg/kg, indicating a potential environmental concern and highlighting elevated pollution in this area [24]. In urban areas, the median concentration of pyrene in surface soils varies greatly, highlighting the impact of population density and industrial activities on contamination levels. Pyrene concentrations are notably low in Tallinn (117 w g/kg) and Vilnius (127 w g/kg), moderately higher in Helsinki (539 w g/kg), but are significantly elevated in Chicago (3263 w g/kg) and London (1728 w g/kg). This substantial variation underscores how larger and more industrialized cities, such as Chicago and London, experience higher pyrene contamination, likely due to increased industrial emissions and vehicular activity [25]. During the summer, dibenz[a,h]anthracene levels in Zhiwu Park at 1.469 mg/kg and Toutunhe Park at 0.615 mg/kg exceed the 0.55 mg/kg screening value, suggesting contamination with potential environmental and health risks [24]. A study analyzed PAH contamination in soils across 35 locations, measuring 16 PAHs. The total PAH concentrations ranged from 0.36 to 11.49 mg/kg, with an average of 1.99 mg/kg, surpassing the permissible limit of 1 mg/kg. Among the PAHs, pyrene had the highest mean concentration at 0.28 mg/kg, followed by naphthalene and acenaphthylene, while phenanthrene had the lowest at 0.06 mg/kg [26]. Research on PAH contamination in agricultural soils of the Pearl River Delta Region, China, indicates that the most prevalent PAHs are five-ring compounds especially benzo[b]fluoranthene with maximum detection at 843 ng/g, followed by four-ring, six-ring, three-ring, and two-ring PAHs. This hierarchy highlights the predominance of higher-ring PAHs in the soil, which often correlates with sources like combustion processes and industrial activities [27]. A study conducted in Shandong Province, China found that three-ring PAHs were the most dominant, with phenanthrene, acenaphthene, and fluorene being the major compounds, contributing 16.3%, 13.1%, and 10.5% to the total PAH concentration, respectively [28]. In Cluj-Napoca, the primary PAH contaminants identified in the soil were naphthalene, benzo(a)anthracene, and benzo(b)fluoranthene. The average concentrations of these PAHs were as follows: naphthalene at 1.23 μg/kg, benzo(a)anthracene at 1.38 μg/kg, and benzo(b)fluoranthene at 1.71 μg/kg. The total PAH content in the soil averaged 7.03 μg/kg [26]. The distribution of PAHs in Taiyuan soil in Northern China reveals that Nap is the most widespread contaminant at 28.39%, while BgP is the least at 0.65%. BbF makes up 28.19% of carcinogenic PAHs, with DBA representing only 0.46%. High-ring PAHs account for 53.51% of total PAHs, low-ring PAHs for 23.99%, and medium-ring PAHs are the least prevalent [27]. Another study conducted in Taiyuan has found that anthracene rose 5.36-fold while in 2016, and acenaphthylene increased 3.37-fold. This increase was more pronounced for pyrogenic PAHs due to continuous fossil fuel emissions and elevated CO_2_ levels. Phenanthrene had the highest concentration, ranging from 43.30 μg kg^−1^ in 2015 to 46.27 μg kg^−1^ in 2016 [21]. A typical chemical industrial park in Chongqing has demonstrated detection rates of phenanthrene (94.44%), fluoranthene (83.33%), and pyrene (83.33%). The primary sources of PAHs in the study area are likely coal combustion and transportation emissions, based on the high percentages of four-ring and five-ring PAHs [29]. A study has also reported that four-ring PAHs such as Kingtom (KT) (698–1750 ng g^−1^ dw) are the most concentrated, followed by five-ring and two-ring PAHs. KT was higher in some urban and rural areas globally, but lower in other high-PAH regions like Beijing and London [30]. On the other hand, in soils of an industrial area in semi-arid Uzbekistan [31], an average concentration of 21.53 ng/g was reported in top-layer soil, where phenanthrene was the most prevalent PAH, contributing 4.25–41.03% of the total PAHs, followed by chrysene at 3.4–24.1%; however PAH concentrations decreased progressively from the surface to deeper soil layers, likely due to atmospheric sedimentation affecting the topsoil more directly. Meanwhile, PAH profiles of agricultural soils of Hamedan County, in the west of Iran indicate that PAHs with four or more rings account for 78% of the total mass of the 16 PAHs that were predominant [32]. Interestingly, a study has found the presence of three-ring PAHs, with phenanthrene (PHE) and 9-fluorenone (9-FO) being the most abundant individual PAH and OPAH, respectively, in soil and vegetation samples from the Antarctic, Arctic, and Tibetan Plateau. Antarctic samples showed higher ratios of PAH derivatives such as oxygenated-PAHs (OPAHs) and nitrated-PAHs (NPAHs) to parent PAHs suggesting increased secondary OPAH and NPAH formation in the region [33]. Increased amounts of PAHs were identified in soil samples from Al-Ahdab oil field in the Waset Region, Iraq, in which fluorene and phenanthrene ranged from 19 to 855 μg/kg dry weight, with a mean concentration of 320 μg/kg [34]. The top three PAHs identified in the soil surrounding the Nam Son landfill area are naphthalene with an average concentration of 10.33 ng/g (range: nd–26.16 ng/g), phenanthrene with an average concentration of 8.43 ng/g (range: 3.16–14.53 ng/g), and benzo[b]fluoranthene with an average concentration of 6.63 ng/g (range: 0.60–30.94 ng/g). The PAHs in this area were primarily attributed to biomass burning (such as straw, grass, and trees) and partially to the combustion of fossil fuels due to agricultural machinery and vehicles [35]. Table 1 provides a summary of the top PAHs and their concentrations from various studies.

PAHs in soil pose significant risks to human health through various exposure routes, including ingestion, dermal contact, inhalation, and bioaccumulation in the food chain. Soil, as a major reservoir of PAHs, presents a more substantial exposure risk than air or water. When PAHs are present in soil, they can enter the human body through direct contact with contaminated soil, consumption of food grown in such soil, or inhalation of soil particles that may contain PAHs [11,12,13]. Over time, PAHs can accumulate in the body, leading to chronic health issues such as cancer, respiratory problems, and reproductive effects. The higher potential for human exposure through soil underscores the critical need for public health interventions to address soil contamination [36]. On the other hand, coastal regions with heavy maritime traffic are especially at risk of PAH pollution because of heavy human activities like industrialization, tourism, and inadequate waste disposal which lead to higher atmospheric PAH levels due to ship emissions and harbor activities. PAHs are released into the air and can settle on water and soil, leading to localized contamination. These PAHs deposited from the atmosphere can accumulate in coastal sediments and aquatic organisms, affecting marine life and potentially entering the food chain. This is particularly relevant in harbors and coastal areas, where pollution sources are concentrated [37,38]. These actions lead to a high level of PAH pollution in estuarine and coastal ecosystems (ECEs), where pollutants move from rivers to bays, worsening contamination in calm areas [39]. The transfer of PAHs from rivers to coastal areas and bays, acting as secondary sources, may result in sediment build up, forming possible contamination zones. Various pathways, such as wastewater discharge, atmospheric deposition, surface runoff, and crude oil leakage, can introduce pollutants into the marine environment [18]. A study has reported that the Arctic Ocean is no longer pristine, and that this is because of climate change, which contributes to the transport of pollutants, making the Arctic a final sink and potential secondary source [40]. Elevated levels of PAHs in soil can have notable effects on ecosystem health by changing soil microbial communities, impacting plant growth and nutrient cycling, and potentially disturbing ecosystem functions and biodiversity. Previous studies have focused on single pollutants’ impacts on microbial communities, with findings showing that PAHs can affect microbial abundance, diversity, and metabolic functions [41,42,43].

## 4. Impact of PAHs on Animals, Humans and Environment

Since these PAHs are hydrophobic in nature, they tend to stick strongly to soil organic matter and black carbon. This characteristic causes soil to be a major storage area for these pollutants [44,45]. The hydrophobicity of PAHs makes them less likely to dissolve in water, leading them to associate with non-polar substances like organic matter present in the soil [46]. Meanwhile, soil’s complex structure and composition, which includes organic matter and clay particles, also plays a significant role in the adsorption and retention of PAHs [47]. Organic matter in soil, such as decomposed plant and animal residues, provides numerous binding sites for PAHs, enhancing their retention. Similarly, clay particles, with their high surface area and charge properties, can adsorb PAH molecules effectively [48]. This high retention capacity results in prolonged environmental persistence of PAHs in soil. The strong binding between PAHs and soil components makes it difficult for these pollutants to be removed or degraded, leading to their accumulation over time [49]. Furthermore, PAHs can be gradually released from the soil into the environment through processes such as leaching, volatilization, or bioturbation [50]. This slow release can lead to continuous exposure of the ecosystem to PAHs, posing long-term environmental, food chain, animal, and health risks [51,52]. 

PAHs enter the food chain through multiple pathways, beginning with their absorption by primary producers such as plants and phytoplankton in aquatic environments. These organisms accumulate PAHs from contaminated soil or water, initiating their transfer into terrestrial and aquatic food webs [53]. Additionally, many animals ingest PAHs inadvertently while consuming soil or sediment, further facilitating their entry into the food chain; for instance, following an explosion at the Baghjan-5 well in May 2020, a long-lasting fire broke out and led to the discharge of natural gas, condensate, and harmful chemicals into the nearby region. This incident severely affected nearby wildlife sanctuaries and rivers [54]. Predation then allows PAHs to move up the trophic levels as contaminated organisms are consumed by predators. Biomagnification of PAHs within food chains exacerbates their environmental impact. This phenomenon results from slow metabolism and excretion rates in many animals, particularly those higher up the food chain. Top predators such as birds of prey, large fish, and marine mammals consequently accumulate higher concentrations of PAHs compared to their prey species [54,55]. Marine animals take in PAHs mainly through their gills or by eating food that contains soluble and particulate fractions, with absorption rates influenced by factors like bioavailability and physiological condition. However, vertebrates typically metabolize and eliminate PAHs more effectively than invertebrates, which lowers the potential for bioaccumulation and biomagnification in marine food chains [56]. Studies have shown that PAHs bioaccumulate in avian tissues, with detectable concentrations found in organs such as the liver, kidneys, lungs, brain, eggs, muscle tissue, blood, and even feces across various bird species [57]. 

PAHs in animals decrease immune function, making the animals more vulnerable to illnesses and parasites [58]. The long-term exposure of animals to PAHs can lead to a range of health issues such as cancer, particularly in the lungs, liver, and skin. Chronic exposure can also lead to respiratory problems such as bronchitis and chronic obstructive pulmonary disease (COPD), as well as liver and kidney damage, manifesting as jaundice and impaired organ function. Additionally, PAHs can cause dermatological issues, and reproductive and developmental toxicity, potentially leading to reduced fertility and developmental delays in offspring [59,60]. PAHs present a substantial health hazard to animals and humans through different avenues of exposure. PAHs are associated with a wide range of health issues due to their toxic, mutagenic, and carcinogenic properties. Naphthalene, one of the most common PAHs, is particularly hazardous, leading to hemolysis (the breakdown of red blood cells) when inhaled or ingested in significant amounts [61]. Additionally, PAHs like benzo[a]pyrene are metabolized into reactive intermediates that bind to DNA, causing mutations that can initiate and promote cancer development [62]. Benzo[a]pyrene is recognized as a Group 1 carcinogen by the International Agency for Research on Cancer (IARC), highlighting its strong association with various cancers, particularly lung, breast, childhood cancers, cervical, and bladder cancers. Other PAHs, like dibenzo[al]pyrene, show higher genotoxicity in experimental settings but are less studied in humans, thus classified in Group 2A (probably carcinogenic) [63]. 

PAHs cause DNA damage primarily by forming DNA adducts. This process begins with the metabolic activation of PAHs, a critical step mediated by cytochrome P450 enzymes, particularly CYP1A1, CYP1A2, and CYP1B1. The activation involves the conversion of PAHs into reactive intermediates that can covalently bind to DNA, forming adducts that can lead to mutations and initiate carcinogenesis. The aryl hydrocarbon receptor (AhR) plays a crucial role in regulating the expression of these cytochrome P450 enzymes, thereby influencing the extent of PAH-induced DNA damage [64,65]. PAH metabolism can generate reactive oxygen species (ROS), leading to oxidative stress and damage to cellular components, including DNA, proteins, and lipids, which may contribute to carcinogenesis [66,67]. PAHs can also induce epigenetic changes, such as DNA methylation and histone modification, which can alter gene expression and contribute to the development and progression of cancer [68]. Inhalation of PAHs is linked to respiratory issues, including bronchitis, asthma, and COPD, as well as an increased risk of cardiovascular diseases due to the oxidative stress and inflammation they cause [69]. Both male and female reproductive systems can be affected by PAH exposure, leading to decreased fertility, hormonal imbalances, and complications during pregnancy [70]. Emerging evidence suggests that PAHs may have neurotoxic effects, contributing to cognitive impairments and neurodegenerative diseases. Furthermore, they can suppress the immune system, increasing susceptibility to infections and exacerbating cancer risk [71]. Vulnerable populations, such as children, the elderly, and those with preexisting conditions, are particularly at risk from PAH exposure. The diverse health effects such as impacts on immune, genetic, developmental, respiratory, and neurological systems emphasize the urgent need to reduce PAH exposure from environmental sources and contaminated food, stressing the importance of thorough approaches in managing air quality, controlling industrial emissions, and ensuring food safety [72,73]. 

Overall, PAH contamination has profound ecological impacts across various environments. These pollutants persist in soil, adversely affecting soil microorganisms, reducing fertility, and inhibiting plant growth and community structures. In aquatic ecosystems, PAHs harm fish, invertebrates, and algae, accumulating in sediments and posing long-term contamination risks [74]. As PAHs bioaccumulate up the food chain, they disproportionately impact predator species, potentially disrupting ecosystem balance and biodiversity [75]. In marine environments, PAHs threaten sensitive ecosystems like coral reefs where herbivory and predation play a crucial role in PAH accumulation in corals, with longer food chains leading to higher bioaccumulation. Despite significant accumulation, PAH exposure does not appear to affect coral symbionts’ photosynthetic efficiency, suggesting that short-term phenanthrene accumulation may not have immediate toxic effects on coral health [76]. Overall, PAH contamination extends beyond direct human health concerns, exerting far-reaching ecological consequences that affect ecosystem stability and biodiversity. Effective management strategies, including long-term monitoring and remediation plans, are crucial to mitigate the ongoing impact of PAH contamination on both environmental and human health scales.

## 5. Pathways of PAH Entry into Plants

Soil contamination by PAHs can significantly impact plants by PAHs being absorbed and transported within the plant system [77,78]. Plants can absorb PAHs from soil via root uptake, stomatal uptake, cuticular penetration, and hydroponic uptake. Root uptake involves the diffusion of PAH molecules through the soil solution into root cells, where they may undergo transformations or be transported within the plant [79,80]. Stomatal uptake occurs when gaseous PAH molecules diffuse through stomatal openings on leaves and into internal leaf tissues [81]. Cuticular penetration involves PAHs diffusing through the waxy cuticle covering plant aerial parts and accumulating in leaf tissues. In hydroponic uptake, plants in controlled environments can absorb PAHs dissolved in water directly from hydroponic solutions [82]. Some studies indicate higher PAH levels in vegetables grown in polluted soil. However, research shows that PAH transport from roots to shoots has minimal impact on shoot PAH accumulation [77]. Instead, gaseous PAHs in the atmosphere are a major source of PAHs in vegetable leaves, suggesting that vegetables from heavily polluted environments may have higher PAH levels [79,80,81,82].

**Figure 1:**
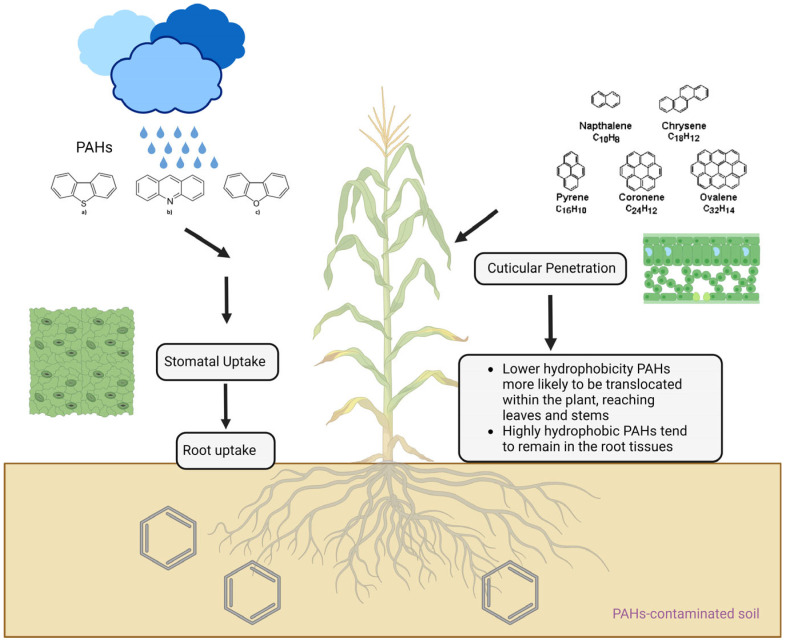
Illustration depicting the uptake and translocation of PAHs within a plant system. The image visualizes the various pathways of PAH absorption and their movement within the plant based on their hydrophobicity.

The uptake and translocation of PAHs within plants depend significantly on their hydrophobicity, often quantified by the octanol–water partition coefficient (log Kow). PAHs with lower log Kow values (<4) are relatively more hydrophilic and can be translocated more readily within the plant, potentially reaching aerial parts like leaves and stems. These compounds can volatilize from contaminated soil or air and be absorbed through the plant cuticle or stomata. In contrast, PAHs with higher log Kow values (>4) tend to remain bound in the root epidermis or outer root tissues, with limited translocation to aerial plant parts [82,83,84]. The uptake of PAHs by plants is influenced by factors such as PAH properties, soil characteristics, plant species, and environmental conditions [85]. Understanding these pathways is crucial for assessing and mitigating PAH contamination in plants and their impact on ecosystems and human health. Table 2 displays the different uptake pathways of PAHs by plants. Figure 1 shows uptake and translocation of PAHs within a plant.

## 6. Impact of PAHs in Plants

PAH pollution is seen as a type of abiotic stress on plants, characterized as a non-living element in the environment that hinders plant growth and progress. PAH stress, unlike natural abiotic stressors such as drought or temperature changes, is usually caused by human activities like industrial operations, urban runoff, or oil spills. This stress can cause different physiological effects like oxidative stress by increasing the generation of ROS, overpowering the plant’s antioxidant mechanisms. PAHs can have various adverse effects, for instance leading to stunting of growth or chlorosis (yellowing of leaves) and necrosis (tissue death) and reduced biomass production [86]. The generation of excessive ROS by PAHs within plant cells can lead to lipid peroxidation, protein oxidation, and DNA damage [87]. Specifically, during times of stress conditions, mitochondria produce ROS through the electron transport chain. Peroxisomes play a role in generating ROS during photorespiration and the breakdown of fatty acids through β-oxidation [88]. Moreover, NADPH oxidases located on the plasma membrane have the ability to produce superoxide when exposed to different types of stress. Overall, oxidative stress impairs cell functionality and compromises plant growth and development [89]. A study has reported elevated superoxide dismutase (SOD) activity, and increased transcript levels of catalase (CAT) and ascorbate peroxidase (APX) in barley leaves upon exposure to biochar, indicating ROS-induced stress. In spite of plants’ antioxidant defense mechanisms, these defenses can be overwhelmed in cases of prolonged exposure to PAHs, hence causing extreme stress and damage [90,91]. 

The interaction between PAHs with plant DNA may create conditions under which mutations in the DNA occur or chromosomal aberrations emerge as well as other genotoxic impacts on plants [92]. Hu et al. have reported that PAHs disrupted rice biomass by affecting light and dark reactions in photosynthesis. Photon absorption and transfer were hindered, and chlorophyll metabolism was disrupted, leading to decreased biosynthesis and increased degradation in roots and leaves. The primary mechanism was the inhibition of chlorophyll a synthesis [93]. Zhang et al. further confirmed that rice plants grown in soil spiked with PAHs (phenanthrene, pyrene, benzo[a]pyrene) exhibited inhibition carboxylation and oxygenation due to the down-regulation of the Rubisco coding gene (OsRBCS2) [94]. In another study involving rice, three pollutants (4′-OH-CB 61, TRI, and BDE 47) also potentially competed with CO_2_ for binding to Rubisco’s active sites, leading to reduction of CO_2_ capture efficiency and a decreased biomass yield [95]. PAH compounds can also dysregulate plant hormonal signaling pathways, leading to abnormal development and physiology. This interference with growth hormones and responses to environmental stresses can disrupt metabolic pathways, affecting the synthesis of secondary metabolites, hormones, and signaling molecules [96]. Genotoxic PAHs can damage DNA, causing mutations or chromosomal aberrations with long-term consequences. Plants can accumulate PAHs from polluted soil, aiding in the bioaccumulation of PAHs through food chains [97]. For instance, distribution and accumulation of PAHs has been observed in winter wheat near a coal-fired power plant where PAH levels were higher in rhizosphere soil, roots, stems, and leaves at the regreening stage compared to the maturity stage. Specifically, root concentration factors (RCF) for three- and four-ring PAHs increased over time, while five-ring PAHs decreased. In stems, three- and four-ring PAHs showed a decreasing trend, while five- and six-ring PAHs initially decreased before stabilizing. This indicates dynamic changes in PAH accumulation and distribution throughout the growth cycle of winter wheat [98]. Leaves act as sensitive air samplers, showing higher concentrations of particle-bound PAHs (H-PAH) concentrations, while lower molecular weight PAHs (L-PAHs) decrease. Vegetation may effectively remove H-PAHs due to their lipophilicity. Pine needles accumulate both L-PAHs and H-PAHs. PAH concentrations are generally higher in pine needles, reflecting their greater surface area. Equilibrium processes govern PAH partitioning between the atmosphere and plants [99]. Table 3 summarizes the various stress responses induced by PAHs in plants.

## 7. Plant Derived Extracellular Vesicles (PDEVs)

Plant derived extracellular vesicles (PDEVs) have shown potential in enhancing plant resilience to PAHs by transporting antioxidants, signaling proteins, and stress-related molecules through facilitating intercellular communication within plants [100]. PDEVs are also integral to plant defense mechanisms. Recent studies have demonstrated the therapeutic potential of PDEVs beyond plant health [101]. PDEVs have shown promising effects in combating inflammation, cancer, bacterial infections, and aging in other biological systems [102]. However, research on PDEVs is growing, with studies exploring their biogenesis, composition, and functions in various plant species. PDEVs have become popular because they are easily accessible, cheap, have low immunogenicity, and are relatively safe in comparison to mammalian-derived exosomes [101]. Research on PDEVs has grown since their discovery in plant cell walls in 1967 [103], as well as the extraction of exosome-like nanoparticles from sunflower seeds in 2009 [104]. PDEVs have been extracted from a range of plant sources, such as fruits, vegetables, seeds, rhizomes, leaves, flowers, bark, tea, and nuts [101]. 

Extracellular vesicles (EVs) derived from plants consist of a variety of vesicles that come from different parts of cells, such as multivesicular bodies (MVBs), autophagosomes, vacuoles, and exocyst-positive organelles (EXPOs) [100]. Similar to mammalian EVs, PDEVs originating from MVBs fuse with the plasma membrane to release EVs outside of the cell wall. EXPO structures, characterized by spherical double membranes, fuse with the plasma membrane to release membrane vesicles. This pathway is unique to plants. Meanwhile, the central vacuole fuses with the plasma membrane, releasing vacuolar proteins and hydrolases into the extracellular compartment to combat extracellular bacterial pathogens. This pathway is also considered a potential source of PDEVs. PDEVs can range from 30 nm to 500 nm, which encompasses the typical sizes of exosomes (around 50–150 nm) and the larger EXPO-derived EVs (200–500 nm) [105]. PDEVs from certain plants like carrots can reach up to 1500 nm in size. This highlights the incredible diversity and variability in the dimensions of these vesicles across different plant species [106]. However, large EVs, in the range of 1–1.5 μm, would likely not fall under the conventional categories of exosomes or microvesicles, which are typically smaller than 1 micrometer [107]. The larger PDEVs may signify unique subtypes or arise from biogenesis pathways that are not fully elucidated [108]. 

The characterization of PDEVs includes assessing particle size distribution, zeta potential, and morphology. PDEVs typically have a zeta potential ranging from −70 mV to neutral [109]. Biochemical analysis has revealed that PDEVs contain bioactive molecules that mediate metabolic and signaling pathways in target cells [110]. However, PDEVs lack established surface protein markers like CD63, CD9, and CD81. Instead, they are enriched in proteins such as aquaporins, heat shock proteins, metabolic enzymes, and annexins [109]. Biomarkers like PENETRATION1 and tetraspanins (such as tetraspanin 8 and tetraspanin 9) have been discovered in EVs from plants, assisting in their identification and monitoring [110]. The lipid makeup of EV membranes from plants, which consists mainly of phosphatidic acid, phosphatidylcholine, digalactosyl diacylglycerol, monogalactosyldiacylglycerol, and phytosterols, is essential for stability, vesicle release, cell-to-cell signaling, and membrane fusion processes [111]. PDEVs contain small RNAs, such as ty RNA, lncRNA, circRNA, and sRNA, which regulate different biological functions and aid in inter-kingdom communication [112].

## 8. Role of Plant EVs in PAH-Induced Stress Response

The stress responses induced by PAHs in plants, as mentioned earlier, are closely related to the potential effects on PDEVs [113,114]. Although research in this specific area is still limited, several potential links between PAH-induced stress and alterations in plant EVs can be identified. The production and release of EVs are closely linked to cellular stress responses. Oxidative stress, membrane damage, and signaling disruptions caused by PAHs could potentially affect the biogenesis and release mechanisms of EVs in plant cells [115]. The cargo (proteins, lipids, nucleic acids) packaged into EVs can reflect the cellular stress state [116]. PAH-induced stress responses, such as the upregulation of antioxidant enzymes, changes in protein synthesis, and alterations in signaling pathways, may lead to changes in the composition of EVs released by plants [117]. Plant cells may utilize EVs as a means of intercellular communication and stress response by selectively loading them with stress-related molecules, such as antioxidants, signaling proteins, or small RNAs [118]. These EVs could potentially be involved in mediating defense mechanisms against PAH-induced stress. EVs have been proposed to play a role in the transport and detoxification of xenobiotic compounds, including PAHs. Plant cells may package and secrete PAHs or their metabolites into EVs as a defense mechanism to remove these toxic substances from the cells [119]. PAHs can disrupt plant cell membranes, potentially affecting the lipid composition and membrane properties of EVs derived from these cells [120]. Changes in EV membrane composition could impact their stability, targeting, and interactions with recipient cells. EVs are involved in intercellular communication and can transfer signals between cells [121]. PAH-induced stress responses may lead to alterations in the signaling cargo or the release and uptake mechanisms of EVs, potentially disrupting their role in stress-related signaling pathways [122,123,124]. While the exact ways PAHs and plant EVs interact are being studied, it is clear that EVs are vital in plant stress responses and may be important for protecting against PAH-related harm. Comprehending these links could offer understanding of plant stress resilience mechanisms and result in the creation of new methods for observing and reducing PAH-triggered stress in plants. Table 4 displays the potential links between PAH-induced stress and alterations in PDEVs.

## 9. Mitigation Strategies and Practical Applications

Plants use a diverse range of methods to reduce the harmful impacts of PAHs by utilizing different biochemical, physiological, and molecular mechanisms such as cuticle formation, unsaturated fatty acids, scavenging reactive species, molecular chaperones, and compatible solutes [122,123,124,125,126,127]. Exposure to PAHs causes plants to prioritize defense and stress management over growth by reallocating energy towards producing defensive compounds and upregulating stress-response proteins (late embryogenesis abundant proteins and their subgroup, dehydrins). This trade-off aids in coping with immediate stress but can lead to stunted growth, reduced root development, and metabolic adjustments to support the production of defense compounds and stress-related proteins [128].

The cuticle serves as a barrier that may restrict the absorption of PAHs from the soil into plant tissues. Moreover, the build-up of wax on the outer layer of the plant has been linked to its ability to handle stress, potentially reducing the negative impacts of exposure to PAHs [125]. Moreover, exposure to PAHs may alter the function of unsaturated fatty acids by disrupting membrane fluidity. Plants have the ability to modify their membrane composition in order to preserve integrity and functionality when faced with PAH stress, just like they do in response to other non-living environmental stresses [126]. Proteins like heat shock proteins (HSPs) serve as molecular chaperones and are essential for maintaining protein folding and stability during stressful conditions [129]. Exposure to PAHs may lead to protein damage, while molecular chaperones could help preserve protein integrity and cellular function. Compatible solutes such as sugars, amino acids (proline and glutamate), and polyols (inositol and mannitol) have the ability to stabilize proteins and membranes when facing stressful conditions. They might also be involved in detoxification or protective functions against stress caused by PAHs in plants [130]. In order to fight against the oxidative stress caused by PAHs, plants boost their antioxidant defense systems by elevating the levels of antioxidant enzymes such as SOD, CAT, and glutathione peroxidase, as well as non-enzymatic antioxidants like glutathione, ascorbate, and carotenoids that eliminate ROS [131,132]. Moreover, plants have the ability to isolate and store PAHs, either by trapping them in vacuoles or by attaching them to lignin in cell walls, which lowers their harmful effects [133]. In terms of metabolism, plants increase the activity of detoxification pathways which include cytochrome P450 enzymes, glutathione S-transferases, and UDP-glucosyltransferases to improve the degradation and binding of PAHs [134,135]. This also leads to an increase in the synthesis of secondary metabolites such as flavonoids and phenolics for added protection [136].

In addition, phytoremediation is a promising method of cleaning up the environment that uses plants and their microbiomes to gather, break down, store, or fix PAHs. Rhizoremediation, a specific form of phytoremediation, involves the breakdown of PAHs in the rhizosphere through interactions between plant roots, their exudates, and soil microorganisms [137]. The success of phytoremediation depends on factors like plant selection, environmental parameters (e.g., nutrient status, contaminant concentration, and bioavailability), soil pH, and the composition and activity of plant-associated microbiomes. Plant root exudates stimulate PAH-degrading microbes, enhancing contaminant degradation. Simultaneously, mycorrhizal fungi form symbiotic relationships with plant roots, improving nutrient absorption and increasing plant resistance to PAH stress [138]. Some of the mycorrhizal fungi can also directly degrade or sequester PAHs, such as *Bjerkandera* sp., *Coriolopsis rigida*, *Irpex lacteus*, *Phanerochaete chrysosporium*, *P. sordida*, *Pleurotus ostreatus*, *P. pulmonarius*, and *Trametes versicolor*. The synergistic interaction between plants and their associated microorganisms (both bacteria and fungi) in the rhizosphere creates a powerful system for PAH degradation. Other strategies include phytoextraction, phytostabilization, and phytovolatilization. For PAHs, rhizoremediation is often the most effective approach due to limited PAH uptake and translocation within most plants. This holistic strategy, leveraging both plant and microbial activities, forms the cornerstone of phytoremediation efforts in PAH-contaminated soils [139,140].

**Figure 2:**
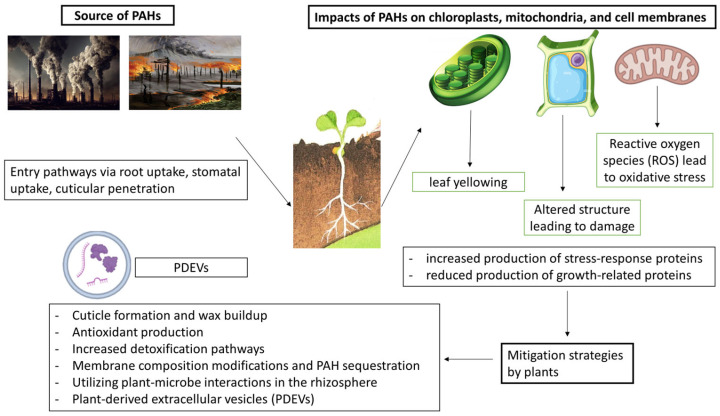
Sources and entry pathways to plant responses and mitigation strategies, and potential role of PDEVs.

Genetic and epigenetic changes in plants help them adapt to PAH stress by increasing stress-responsive gene expression and modifying DNA methylation and histone patterns [141]. This enables plants to effectively combat the adverse effects of PAHs, such as oxidative stress and metabolic disruptions [142]. Exposure to PAHs triggers upregulation of genes encoding for protective molecules like antioxidant enzymes. Additionally, changes in DNA methylation and histone modifications regulate gene expression, enhancing stress responses [143]. These mechanisms collectively boost plant resilience to PAH stress, ensuring their health and productivity in polluted environments. Both genetic and epigenetic factors are crucial for plant stress tolerance and adaptation to PAH pollutants [144]. Figure 2 outlines the key aspects of PAH–plant interactions, from sources and entry pathways to plant responses and potential mitigation strategies, including the role of PDEVs.

## 10. Conclusions

To sum up, PAHs pose significant environmental and health risks due to their persistence and toxicity. Their widespread presence in soil, water, and air ecosystems underscores the urgent need for effective management and remediation strategies. PAHs induce various stress responses in plants, including oxidative stress, disruption of photosynthesis, and alterations in gene expression and metabolism. These effects can significantly impact plant growth, development, and overall ecosystem health. PDEVs have emerged as a promising area of research in the context of PAH-induced stress. These nanoparticles play crucial roles in intercellular communication and stress response, potentially enhancing plant resilience to PAH contamination. The interaction between PAHs and PDEVs, while not fully understood, suggests complex cellular defense mechanisms that may contribute to PAH detoxification and stress mitigation. Phytoremediation, particularly rhizoremediation, offers a sustainable approach to PAH contamination management. This strategy leverages the synergistic interactions between plants, their root exudates, and associated microorganisms to degrade PAHs in the rhizosphere. The success of phytoremediation depends on various factors, including plant selection, environmental parameters, and the composition of plant-associated microbiomes. Future research should focus on elucidating the specific molecular mechanisms of PAH-induced stress in plants and the role of PDEVs in stress resilience. Additionally, developing innovative remediation strategies that integrate plant-based approaches with advanced biotechnological tools could enhance our ability to manage PAH contamination effectively. These efforts are crucial for mitigating the ecological and human health impacts associated with PAH pollution, particularly in vulnerable ecosystems and populations exposed to high levels of these contaminants.

## Figures and Tables

**Table 1:** The top three PAHs and their concentrations for each study location.

Study Location	Top 3 PAHs	Average Concentration	Range
Nam Son Landfill Area	Naphthalene (Nap)	10.33 ng/g	nd–26.16 ng/g
	Phenanthrene (Phe)	8.43 ng/g	3.16–14.53 ng/g
	Benzo[b]fluoranthene (BbF)	6.63 ng/g	0.60–30.94 ng/g
Shandong Province, China	Phenanthrene (Phe)	16.3% of total PAH concentration	-
	Acenaphthene (Ace)	13.1% of total PAH concentration	-
	Fluorene (Fl)	10.5% of total PAH concentration	-
Cluj-Napoca, Romania	Naphthalene (Nap)	1.23 μg/kg	-
	Benzo[a]anthracene (BaA)	1.38 μg/kg	-
	Benzo[b]fluoranthene (BbF)	1.71 μg/kg	-
Taiyuan, Northern China	Naphthalene (Nap)	28.39% of total PAH concentration	-
	Benzo[g,h,i]perylene (BgP)	0.65% of total PAH concentration	-
	Benzo[b]fluoranthene (BbF)	28.19% of total carcinogenic PAHs	-
Zhiwu Park, China	Dibenz[a,h]anthracene (DahN)	1.469 mg/kg	-
Bursa, Turkey	Benzo[a]pyrene (BaP)	-	13–189.4 ng/g dry matter
Antalya Aksu Region, Turkey	Benzo[a]pyrene (BaP)	2.31 μg/kg	-
Uzbekistan (Industrial Area)	Phenanthrene (Phe)	21.53 ng/g	4.25–41.03% of total PAHs
	Chrysene (Chr)	-	3.4–24.1% of total PAHs
Hamedan County, Iran	PAHs with four or more rings	78% of total PAH mass	-
Antarctic, Arctic, Tibetan Plateau	Phenanthrene (Phe)	Most abundant PAH	-

**Table 2:** The uptake pathways of PAHs by plants.

Uptake Pathway	Description	Key Points	References
Root Uptake	Diffusion of PAH molecules through the soil solution into root cells.	PAHs may undergo transformations or be transported within the plant.	[77,78,79,80]
Stomatal Uptake	Diffusion of gaseous PAH molecules through stomatal openings on leaves into internal leaf tissues.	Major source of PAHs in vegetable leaves in polluted environments.	[81]
Cuticular Penetration	Diffusion of PAHs through the waxy cuticle covering plant aerial parts, accumulating in leaf tissues.	Significant for aerial parts of plants in contact with PAH-contaminated environments.	[81]
Hydroponic Uptake	Absorption of PAHs dissolved in water directly from hydroponic solutions in controlled environments.	Used in studies to evaluate PAH uptake in controlled conditions.	[82]

**Table 3:** Summary of the various stress responses induced by PAHs in plants.

Effect of PAHs on Plants	Mechanism	Consequence	References
Oxidative Stress	ROS-induced lipid peroxidation, protein oxidation, and DNA damage	Impaired cell functionality, compromised growth	[89,90,91]
Antioxidant Response	Elevated SOD, CAT, and APX activity	Enhanced defense against oxidative stress	[92,93]
Genotoxicity	Interaction with plant DNA	Mutations, chromosomal aberrations	[94,99]
Photosynthesis Disruption	Inhibition of chlorophyll synthesis, Rubisco down-regulation	Reduced photosynthetic efficiency and biomass yield	[95,96,97]
Hormonal Dysregulation	Interference with hormonal signaling pathways	Abnormal development, disrupted metabolism	[98]

**Table 4:** Summary of potential effects of PAH-induced stress on PDEVs.

Aspect	Potential Effects
Cellular Stress Responses	PAH-induced oxidative stress, membrane damage, and signaling disruptions could affect the production and release mechanisms of plant EVs.
Cargo Composition	PAH-induced stress responses may lead to changes in the composition of EVs, altering the packaging of proteins, lipids, and nucleic acids.
Intercellular Communication	EVs could serve as carriers of stress-related molecules, such as antioxidants, signaling proteins, or small RNAs, facilitating intercellular communication and stress response mediation.
Detoxification Mechanisms	EVs may participate in the detoxification of PAHs by transporting and secreting these compounds or their metabolites as a defense mechanism to remove toxic substances.
Membrane Properties	PAHs can disrupt plant cell membranes, potentially affecting the lipid composition and properties of EV membranes, influencing their stability and interactions with recipient cells.
Role in Signaling Pathways	PAH-induced stress responses might disrupt the release and uptake mechanisms of EVs involved in stress-related signaling pathways, impacting plant stress resilience.

## Data Availability

Data are contained within the article.
